# Supplementary material for: Mesothelin Gene Variants Affect Soluble Mesothelin-Related Protein Levels in the Plasma of Asbestos-Exposed Males and Mesothelioma Patients from Germany
Source: Biology (Basel). 2022 Dec 14;11(12):1826. doi: 10.3390/biology11121826 (PMC9776112; doi:10.3390/biology11121826)
Supplement: Supplementary file 1 [file biology-11-01826-s001.zip › biology-2071895-supplementary.pdf]

Table S1. Logistic regression models in asbestos-exposed controls and mesothelioma (MM) groups adjusted by age.

Model	Variable	Effect	OR	95% CI		P value	AIC
1	Group	Pre-diagnostic MM	**7.98**	**3.00**	**21.20**	**<0.001**	210.98
		Manifest MM	**12.70**	**5.36**	**30.08**	**<0.001**	
		Controls	1				
2	Group	Pre-diagnostic MM	**8.07**	**2.98**	**21.82**	**<0.001**	205.18
		Manifest MM	**17.38**	**6.88**	**43.91**	**<0.001**	
		Controls	1				
	MSLN rs3764247 A>C	Non-common genotype	**3.12**	**1.38**	**7.08**	**0.006**	
		Common genotype (AA)	1				
3	Group	Pre-diagnostic MM	**7.98**	**2.98**	**21.40**	**<0.001**	209.46
		Manifest MM	**15.30**	**6.21**	**37.67**	**<0.001**	
		Controls	1				
	MSLN rs3764246 A>G	Non-common genotype	2.12	0.96	4.70	0.063	
		Common genotype (AA)	1				
4	Group	Pre-diagnostic MM	**8.29**	**3.00**	**22.92**	**<0.001**	201.68
		Manifest MM	**25.78**	**8.96**	**74.24**	**<0.001**	
		Controls	1				
	MSLN rs2235503 C>A	Non-common genotype	**4.77**	**1.84**	**12.36**	**0.001**	
		Common genotype (CC)	1				
5	Group	Pre-diagnostic MM	**8.78**	**3.14**	**24.60**	**<0.001**	200.77
		Manifest MM	**30.99**	**10.19**	**94.27**	**<0.001**	
		Controls	1				
	Haplotype*	3 mutations	**4.86**	**1.73**	**13.66**	**0.003**	
		1-2 mutations	0.56	0.18	1.77	0.325	
		No mutation	1				
6	Group	Pre-diagnostic MM	**7.80**	**2.87**	**21.20**	**<0.001**	204.33
		Manifest MM	**20.46**	**7.72**	**54.21**	**<0.001**	
		Controls	1				
	MSLN rs1057147 G>A	Non-common genotype	**3.51**	**1.48**	**8.30**	**0.004**	
		Common genotype (GG)	1				
7	Group	Pre-diagnostic MM	**10.65**	**3.42**	**33.16**	**<0.001**	203.42
		Manifest MM	**35.09**	**11.06**	**111.29**	**<0.001**	
		Controls	1				
	MSLN rs3764247 A>C	A**C** vs. AA	1.28	0.35	4.73	0.707	
		**CC** vs. AA	1.30	0.18	9.40	0.794	
	MSLN rs3764246 A>G	A**G** vs. AA	0.65	0.18	2.32	0.504	
		**GG** vs. AA	0.97	0.07	12.96	0.980	
	MSLN rs2235503 C>A	C**A** vs. CC	2.27	0.37	14.02	0.378	
		**AA** vs. CC	17.03	0.82	355.39	0.067	
	MSLN rs1057147 G>A	G**A** vs. GG	2.04	0.65	6.35	0.221	
		**AA** vs. GG	1.33	0.25	7.11	0.742	

Model	Variable	Effect	OR	95% CI		P value	AIC
8	Group	Pre-diagnostic MM	**9.11**	**3.13**	**26.48**	**<0.001**	200.06
		Manifest MM	**39.38**	**11.57**	**134.02**	**<0.001**	
		Controls					
	Mutations	1 vs. no mutations	0.40	0.10	1.64	0.205	
		2 vs. no mutations	2.36	0.45	12.25	0.308	
		3 vs. no mutations	0.88	0.16	4.83	0.886	
		4 vs. no mutations	**5.56**	**1.51**	**20.38**	**0.010**	
		5-8 vs. no mutations	**9.78**	**2.61**	**36.62**	**0.001**	

OR Odds ratio; *CI* Confidence interval; *AIC* Akaike Information Criterion

*Haplotype of the three SNPs from 5'-UTR with no mutation (AAC), 1-2 mutations (A**G**C, AA**A**, **C**AC, A**GA**, **C**A**A**, **CG**C), 3 mutations (**CGA**)
